# Predictive value of thrombotic molecular markers combined with venous thromboembolism score for thrombosis in patients with gastrointestinal malignancies

**DOI:** 10.12669/pjms.41.11.12231

**Published:** 2025-11

**Authors:** Yanan Meng, Ji Qi, Yankui Shi, Chengbi Tong

**Affiliations:** 1 Yanan Meng Clinical Laboratory, Affiliated Hospital of Hebei University, Baoding 071000, Hebei, China; 2 Ji Qi Clinical Laboratory, Affiliated Hospital of Hebei University, Baoding 071000, Hebei, China; 3 Yankui Shi, Clinical Laboratory, Affiliated Hospital of Hebei University, Baoding 071000, Hebei, China; 4 Chengbi Tong, Clinical Laboratory, Affiliated Hospital of Hebei University, Baoding 071000, Hebei, China

**Keywords:** Gastrointestinal malignancy, Venous thrombosis, Thrombotic molecular marker, VTE score, Predictive value

## Abstract

**Objective:**

To investigate the predictive value of thrombotic molecular markers, including TAT, PIC, TM, and t-PAIC, combined with the VTE score for venous thrombosis in patients with gastrointestinal malignancies.

**Methodology:**

This was a retrospective study. Two hundred and fifteen patients diagnosed with gastrointestinal malignancies at the Affiliated Hospital of Hebei University between January 2024 and March 2025 were enrolled in this study. Based on whether venous thrombosis occurred during hospitalization after treatment, patients were divided into a thrombosis group(*n=* 34) and a non-thrombosis group(*n=* 181). The levels of thrombotic molecular markers, including TAT, PIC, TM, and t-PAIC, were measured, and the VTE score was recorded for both groups.

**Results:**

The levels of TAT, PIC were significantly higher in the thrombosis group compared to the non-thrombosis group(P<0.05, respectively). ROC curve analysis showed that the area under the curve(AUC) for TAT, PIC, TM, t-PAIC, VTE score, and the integrated model were 0.774, 0.635, 0.539, 0.573, 0.577, and 0.793, respectively. The AUC of the integrated model was significantly higher than that of TAT, PIC, TM, t-PAIC, and the VTE score alone(*P<* 0.05, respectively). At the optimal cutoff value of 0.204, the integrated model achieved a sensitivity of 0.676 and a specificity of 0.851. Multivariate logistic regression analysis identified age, history of cardiovascular disease, and TAT as independent risk factors for VTE (P < 0.05, respectively). Each unit increase in TAT was associated with a 28.9% higher risk of venous thrombosis in patients with gastrointestinal malignancies.

**Conclusion:**

The combination of thrombotic molecular markers and VTE score has significant clinical value in predicting venous thrombosis in patients with gastrointestinal malignancies. This approach facilitates the early identification of high-risk patients.

## INTRODUCTION

Gastrointestinal malignancies, one of the most common types of cancer worldwide, pose a serious threat to human health.^1^ Venous thromboembolism(VTE), a frequent and severe complication in patients with gastrointestinal malignancies, not only increases treatment difficulty and medical costs but also significantly impacts patient prognosis and quality of life.^2^,^3^ Studies have shown that the risk of VTE in patients with gastrointestinal malignancies is significantly higher than in the general population.^4^ The underlying mechanisms involve the biological characteristics of tumor cells, tumor-related treatments, and the hypercoagulable state of the patient. Accurately predicting the risk of thrombosis in these patients is crucial for the early implementation of effective preventive and therapeutic measures.^5^,^6^ Traditional VTE scoring systems play a role in assessing thrombotic risk; however, they have certain limitations.^7^

In recent years, thrombotic molecular markers (TMMs) have garnered increasing attention for their ability to reflect the activation state of the coagulation and fibrinolytic systems, contributing to the diagnosis and risk prediction of thrombotic diseases.^8^ However, the predictive efficacy of a single TMM is limited. Whether combining multiple TMMs with the VTE scoring system can improve the accuracy of thrombosis prediction in patients with gastrointestinal malignancies remains inadequately studied. Therefore, this study aims to investigate the role of TMMs combined with the VTE score in assessing the risk of thrombosis in patients with gastrointestinal malignancies. By comparing baseline characteristics, TMM levels, and the VTE score between the two groups, as well as conducting correlation analysis, ROC curve analysis, and multivariate logistic regression analysis, providing a basis for the early clinical identification of high-risk patients.

## METHODOLOGY

This was a retrospective study. Two hundred and fifteen patients diagnosed with gastrointestinal malignancies at the Affiliated Hospital of Hebei University between January 2024 and March 2025 were enrolled in this study. Patient data were retrieved from electronic medical record systems. A total of 34 patients who developed thrombosis during hospitalization were assigned to the thrombosis group, including 17 males and 17 females, with an age range of 54–79 years and an average age of (67.12 ± 6.80) years. This group included 11 cases of gastric cancer, 10 cases of colon cancer, and 13 cases of rectal cancer. The non-thrombosis group comprised 181 patients, including 112 males and 69 females, with an age range of 30–83 years and an average age of (62.91 ± 10.44) years. This group included 56 cases of gastric cancer, 68 cases of colon cancer, and 57 cases of rectal cancer. General clinical data, including age, sex, height, weight, medical history, tumor type, tumor stage, and treatment modality, were collected. There were no statistically significant differences between the two groups in age, sex, or tumor type composition (all *P >* 0.05).

### Ethical approval:

The study was approved by the Institutional Ethics Committee of Affiliated Hospital of Hebei University (No.: HDFYLL-IIT-2025-030; Date: March 17, 2025).

### Inclusion criteria:

- Diagnosis of gastrointestinal malignancy confirmed by histopathological examination, cytology, or imaging.
- Age ≥18 years.
- Underwent surgical treatment or chemotherapy during hospitalization.

### Exclusion criteria:

- Severe hepatic or renal dysfunction or other severe complications that could affect coagulation test results.
- Use of anticoagulant, antiplatelet, or other coagulation-affecting drugs within the past month.
- Presence of hematologic disorders such as leukemia or thrombocytopenic purpura.
- Pregnancy or lactation.

### TMM Detection:

On the morning of the day following admission, 2.7 mL of fasting venous blood was collected from all patients into vacuum tubes containing 0.109 M sodium citrate as an anticoagulant. The samples were centrifuged at 2060 g for 10 minutes to separate plasma. The levels of thrombin-antithrombin complex (TAT), plasmin-alpha2 plasmin inhibitor complex (PIC), thrombomodulin (TM), and tissue-type plasminogen activator/plasminogen activator inhibitor-I complex (t-PAIC) were measured using chemiluminescence immunoassay on the Wondfo Shine I2900 analyzer, strictly following the manufacturer’s instructions.

### VTE Score:

Assessment the Caprini risk assessment model was used to evaluate the VTE risk score for all patients. This model assigns scores based on multiple factors, including age, sex, underlying diseases, surgical conditions, and tumor-related factors. Risk categories were defined as follows: (1) Low risk: 0–1 points; (2) Moderate risk: 2 points; (3) High risk: 3–4 points; (4) Very high risk: ≥5 points. All assessments were conducted and recorded by physicians who had undergone standardized training.

### Follow-up:

All patients were closely monitored for venous thrombosis during hospitalization after treatment. The diagnosis was confirmed based on clinical manifestations (*e.g*., limb swelling, pain, increased skin temperature, and superficial vein dilation) combined with a color Doppler ultrasound examination. If patients exhibited suspected VTE symptoms, appropriate diagnostic tests were performed promptly, and the location and type of thrombosis were recorded upon confirmation.

### Outcome Measures:

Primary Outcome Measure: Incidence of venous thrombosis during hospitalization after treatment in patients with gastrointestinal malignancies. Secondary Outcome Measures: TMMs (e.g., TAT, PIC, TM, t-PAIC) and the VTE score.

### Statistical analysis:

Statistical analyses were performed using the software SPSS 27.0. Measurement data following a normal distribution were expressed as mean ± standard deviation (*χ̅*±*S*) and compared between groups using an independent samples t-test. Non-normally distributed measurement data were presented as median (interquartile range [IQR]) and analyzed using the rank-sum test. Categorical data were expressed as frequency and percentage (n, %), and between-group comparisons were analyzed using the chi-square (*χ*²) test. The predictive value of individual TMMs and their combination with the VTE score for thrombosis in patients with gastrointestinal malignancies was evaluated using receiver operating characteristic (ROC) curve analysis. The area under the curve (AUC), sensitivity, and specificity were calculated. Additionally, multivariate logistic regression analysis was conducted to identify independent risk factors for VTE. A *P-*value <0.05 was considered statistically significant.

## RESULTS

A comparison of baseline clinical characteristics between the two groups showed statistically significant differences in age, history of cardiovascular disease, and history of thrombosis (*P <* 0.05, respectively) (Table-I). Comparison of TMM levels and the VTE score between the two groups showed statistically significant differences in TAT and PIC levels (*P <* 0.05, respectively) (Table-II).

**Table-I:** Comparison of baseline characteristics between the two groups.

Variable	Thrombosis group (n = 34)	Non-thrombosis group (n = 181)	t/Z/χ² value	P-value
Age	69(61.25,73)	64(57,70)	-2.202	0.028
Height	160(158,170)	165(160,170)	1.268	0.203
Weight	65.81±12.05	64.05±11.26	0.789	0.434
Body mass index	24.6±3.67	23.45±3.54	1.686	0.099
Sex			1.224	0.269
Male	17(50%)	112(61.88%)		
Female	17(50%)	69(38.12%)		
History of cardiovascular disease			7.993	0.005
Absent	27(79.41%)	172(95.03%)		
Present	7(20.59%)	9(4.97%)		
History of diabetes			0.000	1.000
Absent	30(88.24%)	157(86.74%)		
Present	4(11.76%)	24(13.26%)		
History of hypertension			0.035	0.851
Absent	21(61.76%)	118(65.19%)		
Present	13(38.24%)	63(34.81%)		
History of thrombosis			4.612	0.032
Absent	27(79.41%)	168(92.82%)		
Present	7(20.59%)	13(7.18%)		

**Table-II:** Comparison of TMM levels and the VTE score between the two groups.

Variable	Thrombosis group (n = 34)	Non-thrombosis group (n = 181)	Z-value	P-value
TAT	7.68(5.6,9.05)	4(2.53,6.02)	-5.064	<0.001
PIC	0.99(0.68,1.48)	0.71(0.54,1.03)	-2.504	0.012
TM	8.89(7.69,11.12)	8.75(7.19,10.4)	-0.729	0.467
t-PAIC	11.38(7.16,15.1)	8.42(5.63,13.74)	-1.349	0.178
VTE score	5(4.25,6)	5(4,6)	-1.424	0.143

**Fig.1:**
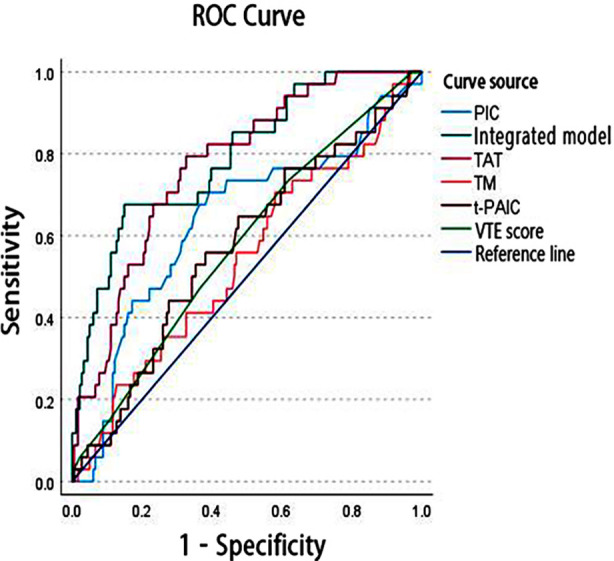
ROC curves of TAT, PIC, TM, t-PAIC alone and combined with the VTE score.

Correlation coefficients range from -1 to 1, where values closer to 1 or -1 indicate a strong correlation, and values near 0 indicate a weak or no correlation. Correlation analysis among TMMs and the VTE score showed statistically significant positive correlations between TAT and PIC, TAT and t-PAIC, TM and t-PAIC, and TM and the VTE score (*P <* 0.05, respectively) (Table-III). ROC curves were plotted for TAT, PIC, TM, and t-PAIC individually, as well as in combination with the VTE score, to predict thrombosis in patients with gastrointestinal malignancies (Fig.1).

**Table-III:** Correlation analysis among TMMs and the VTE score.

Characteristic	TAT	PIC	TM	t-PAIC	VTE score
TAT	1				
PIC	0.250**	1			
TM	0.011	0.129	1		
t-PAIC	0.212**	0.035	0.193**	1	
VTE score	-0.101	-0.011	0.242**	0.05	1

The results showed that the AUC for the integrated model was 0.793, which was significantly higher than that of TAT (AUC = 0.774), PIC (AUC = 0.635), TM (AUC = 0.539), t-PAIC (AUC = 0.573), and the VTE score alone (AUC = 0.577), demonstrating superior predictive accuracy (Table-IV). Additionally, the AUC of the integrated model was significantly higher than that of PIC, TM, t-PAIC, and the VTE score (*P <* 0.05, respectively). At the optimal cutoff value of 0.204, the integrated model achieved a sensitivity of 0.676 and a specificity of 0.851.

**Table-IV:** ROC curve analysis of individual TMMs and the integrated model for predicting thrombosis

Parameter	AUC	95% CI_min_	95% CI_max_	Cutoff value	Sensitivity	Specificity
TAT	0.774	0.695	0.853	0.142	0.794	0.674
PIC	0.635*	0.526	0.745	0.155	0.706	0.608
TM	0.539*	0.430	0.649	0.152	0.756	0.420
t-PAIC	0.573*	0.467	0.679	0.156	0.559	0.619
VTE score	0.577*	0.476	0.679	0.138	0.735	0.381
Integrated model	0.793*	0.710	0.877	0.204	0.676	0.851

***Note:***

* *P <*0.05 compared to the integrated model.

To identify independent risk factors for VTE, the variables with significant differences in baseline clinical characteristics and TMM levels between the two groups were included in a multivariate logistic regression model, namely, age, history of cardiovascular disease, history of thrombosis, TAT, and PIC. The results indicated that age, history of cardiovascular disease, and TAT were independent risk factors for VTE (*P <* 0.05, respectively) (Table-V). For every one-year increase in age, the risk of venous thrombosis in patients with gastrointestinal malignancies increased by 6.7%. Patients with a history of cardiovascular disease had a 3.796-fold higher risk of thrombosis compared to those without. For each unit increase in TAT, the risk of venous thrombosis increased by 28.9%.

**Table-V:** Multivariate Logistic regression analysis of VTE risk factors in patients with gastrointestinal malignancies.

Variable	β-value	Standard error	Wald χ² value	P-value	OR	95% CI
Constant	-7.320	1.918	14.572	<0.001	0.001	
Age	0.065	0.027	5.926	0.015	1.067	1.013-1.125
History of cardiovascular disease (Present vs. Absent)	1.334	0.637	4.380	0.036	3.796	1.088-13.235
History of thrombosis (Present vs. Absent)	1.015	0.590	2.965	0.085	2.76	0.869–8.766
TAT	0.254	0.062	17.065	<0.001	1.289	1.143–1.455
PIC	-0.478	0.283	2.855	0.091	0.62	0.356–1.079

## DISCUSSION

In the comparison of baseline characteristics, we found significant differences in age, history of cardiovascular disease, and history of thrombosis between the thrombosis and non-thrombosis groups. These factors are recognized as important risk factors for thrombosis.^9^ Aging may contribute to thrombosis by causing vascular wall degeneration and alterations in blood rheology, increasing the likelihood of clot formation.^10^ Similarly, a history of cardiovascular disease and thrombosis directly reflect vascular health and past thrombotic events, significantly increasing the risk of recurrent thrombosis. In the comparison of TMM levels and the VTE score, significant differences were observed in TAT and PIC between the two groups. TAT is a key marker in the coagulation process, and elevated TAT levels may indicate enhanced coagulation activity.^11^ PIC (plasmin-α2-antiplasmin complex) is a marker of the fibrinolytic system, and changes in its levels may reflect alterations in fibrinolytic activity. Abnormalities in these molecular markers may be associated with the imbalance between coagulation and fibrinolysis during thrombosis.^12^

Correlation analysis revealed significant positive correlations between TAT and PIC, TAT and t-PAIC, TM and t-PAIC, as well as TM and the VTE score. These findings suggest potential interactions among these molecular markers, indicating their collective involvement in the pathological process of thrombosis.^13^ Notably, the significant positive correlation between TM and the VTE score suggests that TM may play a crucial role in thrombosis.^14^ All these provide fresh insights into the molecular mechanisms underlying thrombosis.^15^

In the ROC curve analysis for thrombosis prediction, we found that the integrated model exhibited significantly higher predictive accuracy than individual markers and the VTE score. This indicates that incorporating multiple molecular markers along with the VTE score provides a more comprehensive risk assessment in patients at risk of thrombosis.^16^ The high sensitivity and specificity of the integrated model make it a promising predictive tool for clinical practice. By determining the optimal cutoff value, high-risk patients can be more accurately identified, thereby supporting clinical decision-making.^17^ Multivariate logistic regression analysis further confirmed that age, history of cardiovascular disease, and TAT are independent risk factors for VTE. The significance of these factors highlights their critical role in the risk assessment for thrombosis.^18^ Notably, for each unit increase in TAT, the likelihood of venous thrombosis increased by nearly 30%, underscoring its importance in clinical risk evaluation.^19^ Additionally, the significantly elevated risk of thrombosis in those with a history of cardiovascular disease also suggests a close relationship between cardiovascular conditions and thrombotic events.^20^

### Limitations

However, a small number of samples is the shortcoming of this study. In view of this, more samples should be included in future studies to further validate the findings of this study. Future research could further explore the role of these molecular markers in different types of malignancies and their potential applications in thrombosis prevention and treatment. Additionally, large-scale prospective studies will be instrumental in validating these findings and refining thrombosis risk assessment models. Through these efforts, we aim to improve the accuracy of thrombosis risk prediction, ultimately enhancing patient care and health outcomes.

## CONCLUSIONS

The current study provides a new perspective and tool for assessing the risk of thrombosis in patients with gastrointestinal malignancies by comprehensively analyzing TMMs combined with the VTE score. These findings not only enhance our understanding of the molecular mechanisms underlying thrombosis but also offer valuable guidance for risk assessment and the development of preventive strategies in clinical practice.

### Authors’ Contributions:

**YM:** Literature search, Conceived and designed the study.

**JQ:** Collected the data and performed the analysis. Critical Review.

**YS** and **CT:** Preparation of the manuscript and is responsible for the integrity of the study.

All authors have read and approved the final manuscript.
